# Adaptation within embryonic and neonatal heart environment reveals alternative fates for adult c‐kit^+^ cardiac interstitial cells

**DOI:** 10.1002/sctm.19-0277

**Published:** 2019-12-31

**Authors:** Bingyan J. Wang, Roberto Alvarez, Alvin Muliono, Sharon Sengphanith, Megan M. Monsanto, Joi Weeks, Roberto Sacripanti, Mark A. Sussman

**Affiliations:** ^1^ SDSU Heart Institute and Department of Biology San Diego State University San Diego California

**Keywords:** adaptation, cardiac, cell culture, heart, interstitial cell

## Abstract

Cardiac interstitial cells (CICs) perform essential roles in myocardial biology through preservation of homeostasis as well as response to injury or stress. Studies of murine CIC biology reveal remarkable plasticity in terms of transcriptional reprogramming and ploidy state with important implications for function. Despite over a decade of characterization and in vivo utilization of adult c‐Kit^+^ CIC (cCIC), adaptability and functional responses upon delivery to adult mammalian hearts remain poorly understood. Limitations of characterizing cCIC biology following in vitro expansion and adoptive transfer into the adult heart were circumvented by delivery of the donated cells into early cardiogenic environments of embryonic, fetal, and early postnatal developing hearts. These three developmental stages were permissive for retention and persistence, enabling phenotypic evaluation of in vitro expanded cCICs after delivery as well as tissue response following introduction to the host environment. Embryonic blastocyst environment prompted cCIC integration into trophectoderm as well as persistence in amniochorionic membrane. Delivery to fetal myocardium yielded cCIC perivascular localization with fibroblast‐like phenotype, similar to cCICs introduced to postnatal P3 heart with persistent cell cycle activity for up to 4 weeks. Fibroblast‐like phenotype of exogenously transferred cCICs in fetal and postnatal cardiogenic environments is consistent with inability to contribute directly toward cardiogenesis and lack of functional integration with host myocardium. In contrast, cCICs incorporation into extra‐embryonic membranes is consistent with fate of polyploid cells in blastocysts. These findings provide insight into cCIC biology, their inherent predisposition toward fibroblast fates in cardiogenic environments, and remarkable participation in extra‐embryonic tissue formation.

## Graphical abstract

Adult c‐Kit^+^cardiac interstitial cells (cCICs) were assessed for retention, persistence, and phenotypic characteristics at three developmental stages: embryonic (E3.5), fetal (E15.5), and neonatal hearts (P3). cCICs delivered into E3.5 subsequently integrated into extra‐embryonic membranes. cCICs delivered into E15.5 and P3 exhibited a fibroblast‐like phenotype, with persistence and cell cycle activity up to 28 days in P3 hearts.

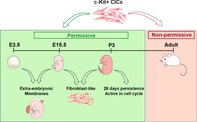

**Significance statement**

Biological properties and functional activities of adult cardiac interstitial cells continue to elude simple characterization despite decades of investigation. The present study demonstrates the influence of developmental environmental cues upon phenotypic properties of c‐Kit^+^ adult cardiac interstitial cells (cCICs). Delivery of cCIC into early embryonic blastocysts leads to trophectoderm integration with exclusion from the inner cell mass, whereas introduction of cCIC into developing myocardium of late fetal or early postnatal hearts results in extended persistence and acquisition of phenotypic traits consistent with fibroblasts. Findings of the present study support the rationale for cCIC cell therapy in the context of congenital and pediatric cardiomyopathic conditions.

**Abbreviations**
AM: amniochorionic membrane
AzG: Azami Green
cCIC: c‐Kit^+^ cardiac interstitial cell
CIC: cardiac interstitial cell
c‐Kit: tyrosine‐protein kinase kit or CD117
dpi: days postinjection
E: embryonic day #
EB: embryoid body
ECM: extracellular matrix
ESC: embryonic stem cell
FUCCI: fluorescence ubiquitination‐based cell cycle indicators
hpi: hours postinjection
ICM: inner cell mass
LV: left ventricle
mKO: monomeric Kusabira Orange
P: postnatal day #
SMA: smooth muscle actin
TE: trophectoderm
TenC: tenascin C
TUNEL: terminal deoxynucleotidyl transferase dUTP nick end labeling
Vim: vimentin

## 1 INTRODUCTION

Myocardial homeostasis is maintained by dynamic interaction on multiple levels between cardiomyocytes and the cardiac interstitial cell (CIC) population. Decades of study reveals CICs as a heterogeneous collection of cell types that defy simple categorization, due in part to their fluid adaptability in response to development, aging, acute injury, and chronic stress.^1^, ^2^, ^3^ Parsing out CIC subtypes with specific markers such as periostin or Tcf21 has merged with the more impartial and nuanced approach of transcriptomic profiling at the single cell level.^4^, ^5^, ^6^ Appreciation for the complexity of CIC biological properties continues to grow, as does recognition that environmental influences exert profound control over CIC phenotypic characteristics and functional activities.

Studies of CIC biology often rely upon assessments performed using populations expanded by in vitro cell culture for various reasons of sample yield, manipulability, and of course simplification compared with challenges of the myocardial milieu in vivo.^7^, ^8^, ^9^, ^10^, ^11^ Such studies provide tremendous insights but also are limited by inescapable aspects of cell culture adaptation, natural selection ex vivo for robust proliferative cell subsets, and multiple choices for conditions of experimental design. Collectively, these variables contribute to the wide range of interpretations and published literature for CIC biology that has been extensively reviewed.^4^, ^12^, ^13^, ^14^ Moreover, a plethora of selected subpopulations of in vitro expanded CICs have been intensively studied for cardioprotective and reparative potential upon reintroduction into pathologically injured myocardium for over a decade,^10^, ^15^, ^16^ but consequences of cell culture environment upon CIC properties in terms of reshaping population characteristics or individual cellular functional capabilities remain relatively unstudied and poorly understood. Typically, such cultures involve two‐dimensional (2D) monolayer growth and serial passaging to obtain sufficient numbers of cells for treatments.^17^, ^18^, ^19^, ^20^ Such 2D culture conditions promote reprogramming toward a common shared transcriptional profile, even between CIC subpopulations enriched by selection for unrelated markers as well as comparisons between multiple donor sources.^5^, ^21^, ^22^ Taken further, our group found that relatively short‐term 2D cell culture for five serial passages results in loss of cell‐specific identity markers and increased homogeneity in a CIC subpopulation enriched for tyrosine‐protein kinase kit or CD117 (c‐Kit^+^) cardiac interstitial cell (cCIC) compared with correspondingly selected freshly isolated cells by single‐cell RNA‐Seq transcriptional profiling.^22^ Findings such as these support the contention that CIC isolation and propagation conditions exert profound influences upon biological and functional properties, consistent with our recent reports of hypoxic culture conditions antagonizing mitochondrial dysfunction and senescence in human cCICs^19^ as well as tetraploid conversion of murine cCICs.^23^ Surprisingly, despite irrefutable evidence of alterations following in vitro expansion of primary CIC isolates, there are essentially no studies to document the extent of such changes as permanent or transient and whether CICs undergo another round of phenotypic and functional adaptation following reintroduction to their native environment of in vivo myocardium.

A major impediment to assessing readaptation of cultured CICs following delivery to host adult myocardium is poor retention and persistence of the donated cell population.^24^, ^25^, ^26^, ^27^ Although using augmented approaches to embed CICs offers some improvement over direct injection to recipient myocardium, bioengineering solutions involving injectable gels or cultured patches severely limits direct interaction between exogenously introduced CICs and host myocardium. Furthermore, delivery to pathologically injured myocardium further stresses the CIC population already coping with dramatic changes in environmental conditions. For example, host immune‐mediated reaction to pathologic injury including CIC delivery prompts a powerful inflammatory response involving cytotoxic action. Indeed, developing myocardium exhibits stage‐specific permissivity for incorporation of introduced or migrating cells.^1^, ^28^ Therefore, we reasoned that assessment of cultured cCIC adaptation following reintroduction to myocardial tissue in vivo would be facilitated by delivery to early developmental stages characterized by cardiogenic activity and negligible inflammation.

Permissive conditions present in embryonic tissue or an early stage developing heart allows for engraftment and persistence of injected cCICs, then followed in subsequent days to weeks for determination of phenotypic characteristics exhibited by both exogenously introduced cells as well as host reaction to their presence. Three distinct embryonic (E3.5), fetal (E15.5), and postnatal (P3) developmental stages were chosen for introduction of cCICs. Results demonstrate engraftment and extended persistence of cCICs including exclusion from the inner cell mass (ICM) of preimplantation blastocysts. Additionally, cCICs display negligible adaptability and functional plasticity following delivery to cardiogenic fetal or postnatal hearts. These findings implicate in vitro expansion as a primary determining factor in cCIC adaptability and provide novel insight regarding cCIC biology.

## 2 RESULTS

### 2.1 Mesodermal potential maintained by cCIC in vitro

cCICs were genetically modified to stably express mCherry fluorescent protein by lentiviral infection, with expanded cCICs exhibiting spindle‐shaped morphology in culture (Figure S1a; 97.6% mCherry^+^). Robust expression of *c‐Myc, Gata3, Gata6*, and *Gata4* mRNAs relative to embryonic stem cells (ESCs) is evident by quantitative PCR (Figure S1b), and cCICs showed the lowest pro‐oncogene expression profile relative to ESC or the whole heart (Figure S1c). Spontaneous aggregation into 3D embryoid body spheres (EBs) in suspension culture is commonly used to study ESC differentiation potential,^11^, ^29^ and culture expanded cCICs similarly aggregate into spheres (Figure S1d). Mesoderm induction treatment of cCIC‐spheres in adherent culture showed increased expression of SM22 alpha (SM22α), whereas endoderm (α‐Fetoprotein, AFP) and ectoderm (βIII‐Tubulin, TUJ1) markers remained undetectable before and after differentiation (Figure S1e). cCICs uniquely express SM22α but not AFP shown by confocal microscopy immunolabeling (Figure S1f), confirming that in vitro expanded cCICs are capable of expressing SM22α^+^. In addition to mesoderm potential, a majority of mesodermal induced cCICs express the fibroblast marker vimentin (Vim), consistent with fibroblast origin (Figure S1g). Collectively, these findings portray cCIC in culture as mesodermal‐lineage derived cells with characteristic fibroblast‐associated marker expression.

### 2.2 Extra‐embryonic tissue integration of cCIC in preimplantation blastocysts

**Figure 1:**
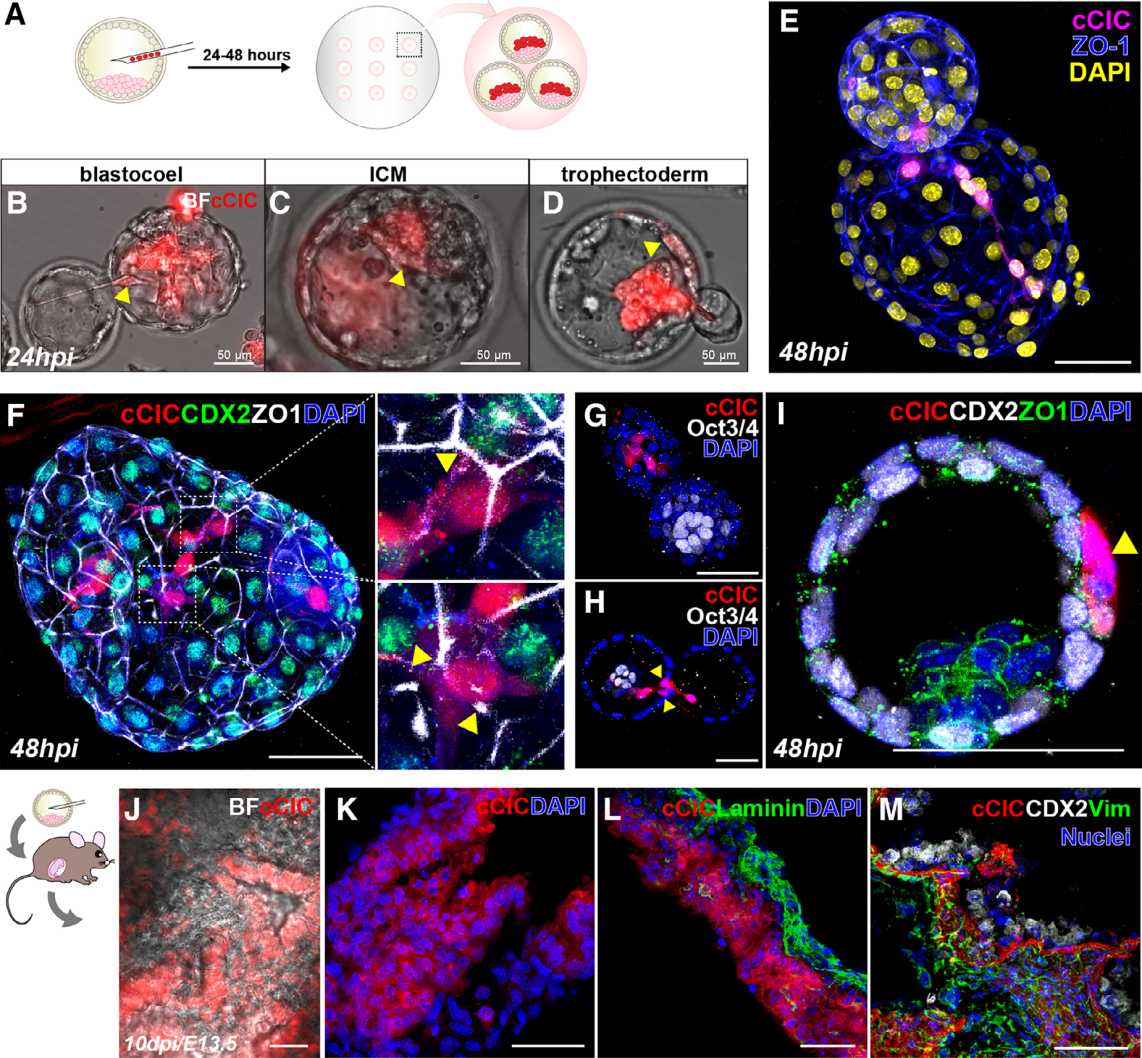
C‐Kit^+^ cardiac interstitial cells (cCICs) integrate into preimplantation blastocysts and adopted extra‐embryonic fate. A, Schematic of blastocyst injection and ex vivo incubation for 24‐48 hours. (b‐d) At 24 hours postinjection (hpi), injected cCICs were retained in blastocoel (B, n = 6/11), inner cell mass (ICM; C, n = 2/11), and trophoblast (D, n = 8/11). See also Video S1. E, At 48 hpi, whole‐mount immunostaining of injected blastocyst showing cCICs anchored with host cells and spread out as spindle morphology in a hatching blastocyst blastocoel. See also Video S2. F, Left, whole‐mount immunostaining of injected blastocyst showing cCICs sharing tight junction (ZO1, white) with host trophectoderm (TE) layer (CDX2, green). Right, higher magnification of boxed area. Arrowheads: ZO1 junctions. G, Immunostaining of ICM marker Oct3/4 (white) showing cCICs do not integrate into ICM. H, A longitudinal optical section showing nuclei (arrowheads) of cCICs located at TE layer. I, Higher magnification of transverse optical section showing cCICs (arrowhead) integrated among nuclei (DAPI, blue) of trophoblasts (CDX2, white), sharing tight junctions (ZO1, green). J, After uterine transfer into pseudopregnant female, cCICs were detected in a mosaic pattern in extra‐embryonic membrane from a chimeric embryo from blastocyst injection at 10 dpi/E13.5. K, Fluorescent scanning of a frozen sectioned extra‐embryonic membrane showing mosaic cCICs integration. Nuclei, DAPI, blue. L, Immunostaining of Laminin showing integrated cCICs localized to the opposite side of epithelial layer of extra‐embryonic tissue. Laminin, green. M, Immunostaining showing cCICs locate in proximity of trophoblast (CDX2, white) and express fibroblast marker (vim, green) in extraembryonic tissue (n = 5). Scale bar, 50 μm

Chimeras blastocyst formation following cell injection is used as a stringent assessment for testing stem cell pluripotency.^30^, ^31^ Adult multipotent cells may harbor properties similar to ESCs allowing for chimera formation when injected into blastocysts.^32^, ^33^, ^34^ Therefore, cCICs were delivered into murine blastocysts that were subsequently cultured ex vivo for 24 to 48 hours postinjection (hpi; Figure 1A). The presence of injected cCICs was directly visualized by expressed mCherry fluorescence without immunolabeling. Injected cCICs persist in the blastocoel, ICM, and trophectoderm (TE) of blastocysts at 24 hpi (Figure 1B‐d, arrowheads, Video S1). Spindle‐shaped morphology of in vitro cCIC (Figure S1a) was observed in hatching blastocysts at 48 hpi (Figure 1E, Video S2). Coupling between cCICs and blastocyst cells is revealed by the presence of tight junctions (Figure 1F, ZO1, arrowheads) shared with neighboring host trophoblasts (CDX2) but rarely with the ICM (Oct3/4) (Figure 1G). cCIC location among the monolayer TE ring immediately adjacent to trophoblasts was visualized by confocal optical sectioning of cCIC nuclei (Figure 1H‐I). cCIC anchoring among trophoblasts in the preimplantation chimeric blastocyst suggests extra‐embryonic tissue integration, assessed by surgical transfer of chimeric blastocysts into pseudopregnant females. Following the anticipated extra‐embryonic pattern, cCICs mosaically integrate predominantly in chorionic lamina of amniochorionic membrane (AM) opposite from squamous amniotic epithelium (Laminin^+^) at 10 days postinjection (dpi; E13.5, Figure 1J‐L). Engrafted cCICs locate adjacent to CDX2^+^ cells and express fibroblast marker vim in extraembryonic tissue (Figure 1M). In contrast, the absence of cCICs from the ICM of developing embryonic tissue was exhaustively evaluated without a single positive finding (n = 253), whereas embryo chimerism was readily observed with a frequency of 19.2% using ESC as a control cell (n = 10/52; Table 1, Figure S2). Therefore, although cCICs possess sufficient functional capacity for extra‐embryonic tissue integration, they are unable to participate in embryonic chimerism.

**Table 1:** Generation of chimeric mice

Cell source	No. of blastocysts transferred	No. of embryos	% Viability	No. of chimeras	% chimerism	Extend of donor cell contribution to embryo proper
cCIC	1055	253	24.0	0	0	None
ESC (control)	123	52	42.3	10	19.2	Heart, epidermis, liver, somites, intestines
Total	1178	305	25.9			

Abbreviations: cCIC, c‐Kit^+^ cardiac interstitial cell; ESC, embryonic stem cell.

### 2.3 Fetal myocardium retains cCIC at perivascular regions

**Figure 2:**
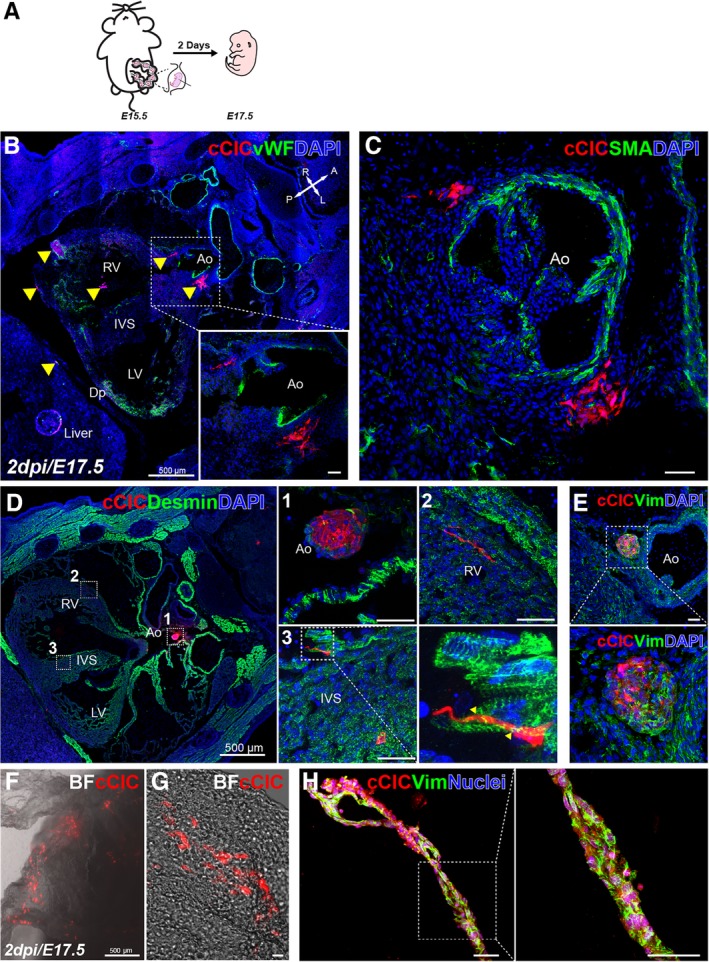
C‐Kit^+^ cardiac interstitial cells (cCICs) maintained fibroblast‐like phenotype and integrated in extra‐embryonic membrane following in utero transplantation (IUT). A, Schematic of IUT in E15.5 embryos and sample collection at E17.5 (2 dpi). B, Clusters of cCICs are scattered in the heart and nearby extracardiac tissues (arrowheads) (n = 4/6). Inset, higher magnification of boxed area. vWF, von Willebrand factor, green. Ao, aorta; LV, left ventricle. IVS, interventricular septum. Cross arrows indicate anatomical axis: A, anterior. P, posterior. L, left. R, right. C, Clusters of cCICs at peri‐aortic valve region. SMA, smooth muscle actin, green. D, Immunostaining of cardiomyocyte lineage marker Desmin, boxed area shown in higher magnification in one Ao, two RV, three IVS. E, Vim staining of a cluster of cCICs showing fibroblast lineage at perivascular region. Vim, Vimentin, green. F and G, cCICs were detected in extra‐embryonic membrane from IUT injected embryo at 2 dpi. BF, bright field. (n = 6/6). H, Immunostaining of cCICs expressing vim (green) (n = 4). Scale bar, 50 μm or as indicated

Empirical testing of in utero transplantation into pericardial space of approximately 5000 cCICs in a time course ranging from E7.5‐E16.5 (data not shown) revealed the optimal prenatal stage for engraftment and persistence was E15.5 (Figure 2A). Assessment of cCIC fate performed 2 days after in utero delivery revealed persistence at multiple intracardial and pericardial locations (Figure 2B, arrowheads), particularly at perivascular regions around tricuspid aortic valve (Figure 2c, Ao). Retained cells were also found in extra‐cardiac tissues within the vicinity of thoracic cavity including thymus, lung, diaphragm, and skeletal muscle (Figure S3a‐e). Embedded cCICs are negative for cardiogenic lineage markers von Willebrand Factor (Figure 2B, Ao), smooth muscle actin (SMA; Figure 2c, Ao), Desmin (Figure 2d, Ao, RV, IVS), and the M‐phase marker phospho‐histone H3 (Figure S3f). However, cCICs in perivascular regions express the fibroblast marker Vim (Figure 2E, green). Consistent with previous observations from blastocyst chimeras (Figure 1J‐K), fetal AM incorporated cCICs in a mosaic pattern with vim expression (Figure 2F‐H), confirming functional capacity and fibroblast cell fate of cCIC contribution to extra‐embryonic tissues. Thus, the prenatal cardiogenic environment allows for engraftment and persistence of injected cCICs that do not contribute directly toward cardiogenesis but instead maintain a fibroblast‐like phenotype.

### 2.4 Neonatal myocardium allows for extended persistence of cCICs

**Figure 3:**
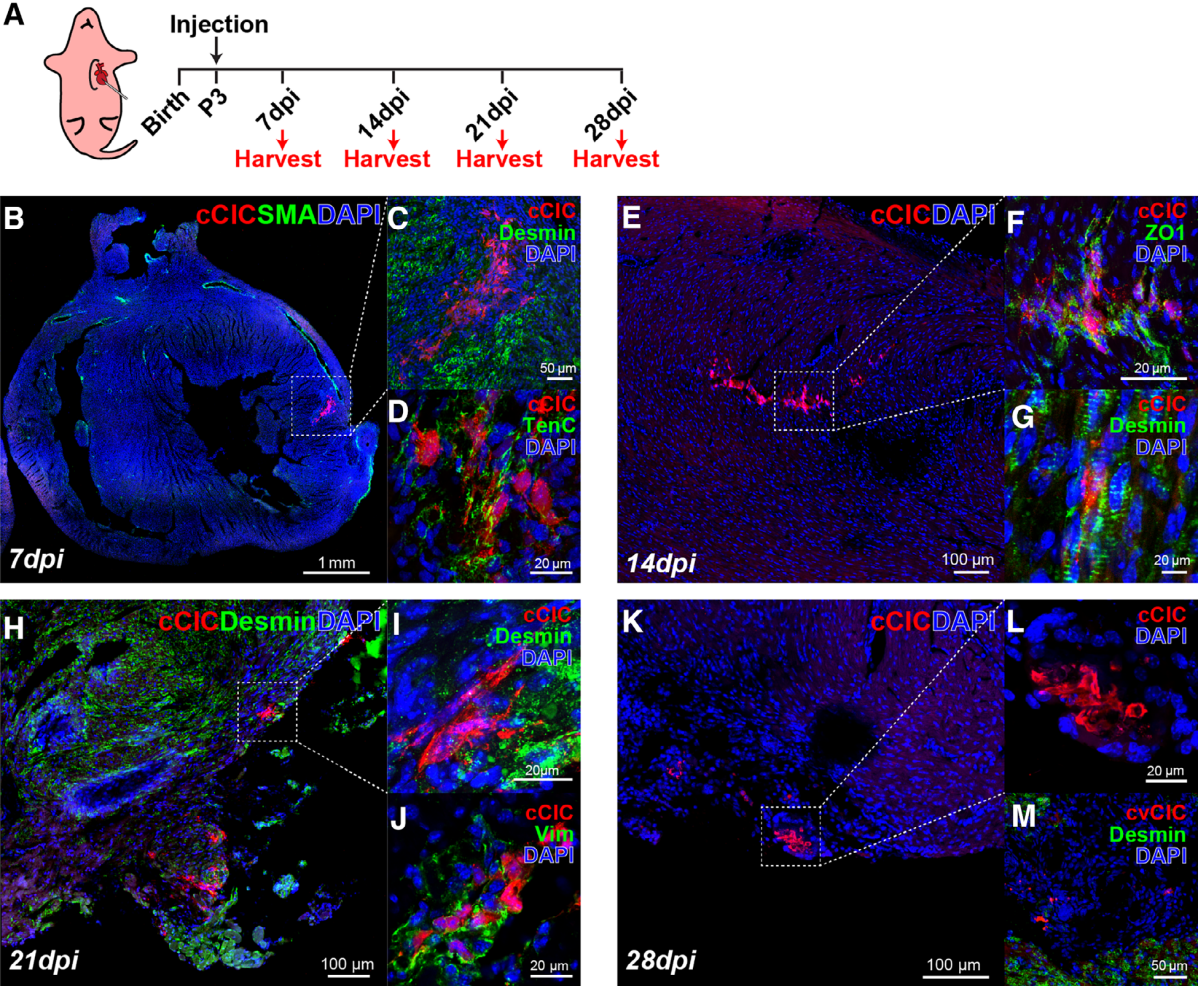
Neonatal myocardium allows for extended persistence of c‐Kit^+^ cardiac interstitial cells (cCICs). A, Schematic of neonatal injection at P3 and sample collection at 7‐day interval for 28 days. B, Tilescan showing cCICs are retained as patches within left ventricular (LV) wall at 7 days postinjection (dpi; n = 5/5). C, Zoomed‐in view of boxed area in (B) showing cCICs do not colocalize with cardiomyocytes (Desmin, green) at 7 dpi. D, cCICs express TenC at early injection period. E, Tilescan showing cCICs are integrated within LV wall at 14 dpi (n = 6/6). F, Zoomed‐in view of boxed area in (E) showing cCICs share tight junctions (ZO1, green) with resident neighboring host cells at 14 dpi. G, cCICs intercalated among resident cardiomyocytes (Desmin, green) at 14 dpi. H, Tilescan of cCICs persistence at LV apex area at 21 dpi (n = 9/16). I, Zoom‐in of boxed area in (H) showing cCICs spindle morphology and closely localized to neighboring cardiomyocytes (Desmin, green). J, cCICs continue to express TenC at 21 dpi. K, Tilescan showing cCICs persist at LV apex area at 28 dpi (n = 3/3). L, Zoomed‐in view of boxed area in (K). M, cCICs do not colocalize with cardiomyocytes (Desmin, green) at 28 dpi

Empirical testing for intramyocardial injection of approximately 5000 cCICs in a time course ranging from P0 to P5 (data not shown) revealed the optimal postnatal stage for engraftment and persistence was P3 (Figure 3A). Assessment of cCIC fate performed every 7 days until 28 dpi revealed several distinct features depending upon the time point examined. Patches of mCherry^+^ cCICs were found within the left ventricular (LV) myocardium at 7 dpi with spindle‐shaped morphology aligned along host myocardium (Figure 3B). Consistent with cCIC phenotype in the fetal heart (Figure 2c‐d), cCICs in the postnatal myocardium lack expression of cardiac lineage markers for SMA or cardiomyocytes (Desmin) at 7 dpi (Figure 3B‐c). Tenascin C (TenC) accumulates in myocardium surrounding persisting cCICs at 7 dpi indicative of extracellular matrix (ECM) remodeling response (Figure 3d). Patches of cCICs remain in LV myocardium at 14 dpi (Figure 3E) that form ZO1‐associated tight junctions with neighboring host myocardium (Figure 3F). Although cCICs intercalate between resident myocytes, the expression of markers for cardiogenic lineage remains absent at 14 dpi (Figure 3G). Following cCIC fate at 21 and 28 dpi showed persistence at the LV apex region, although cell number was diminished relative to levels at 7 and 14 dpi (Figure 3H, K). Endogenous mCherry tag fluorescence grew dim at these later time points, requiring immunolabeling to amplify the signal for confocal imaging. Surviving cCICs maintain proximity to cardiomyocytes as well as fibroblast‐associated Vim expression at 21 dpi (Figure 3I‐J). However, a week later at 28 dpi, the spindle‐shape morphology of remaining cCICs becomes increasingly indistinct as distance from cardiomyocytes increases (Figure 3L‐M). Primary conclusions from postnatal injections of cCICs are (a) remarkable persistence for at least 28 dpi and (b) cell marker expression consistent with fibroblast lineage in the absence of any cardiogenic commitment.

### 2.5 Multiple factors contribute to cCIC persistence in postnatal hearts

**Figure 4:**
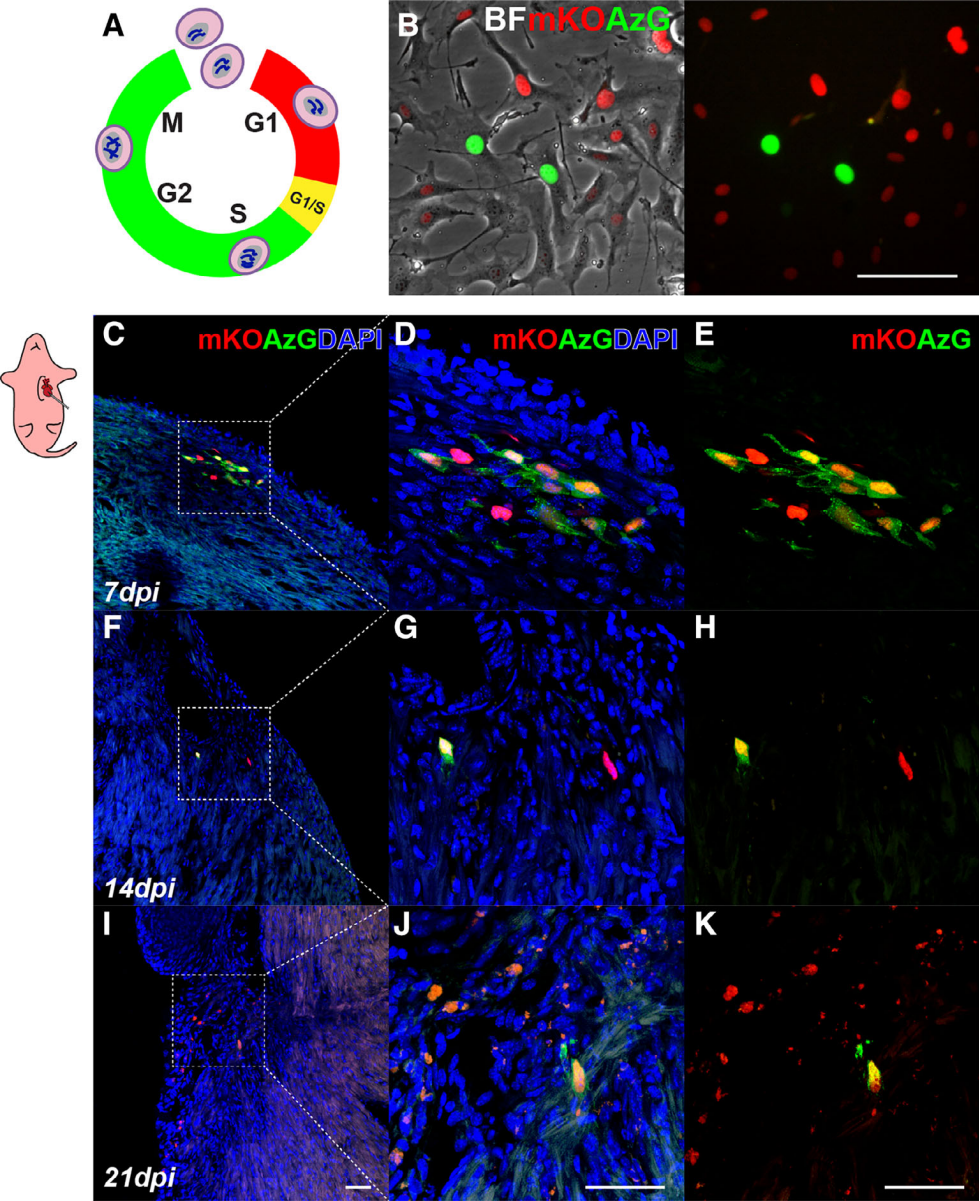
Engrafted c‐Kit^+^ cardiac interstitial cells (cCICs) remain active in cell cycle for up to 14 days revealed by fluorescence ubiquitination‐based cell cycle indicators (FUCCI). A, Schematic of FUCCI fluorescence oscillation and cell cycle progression. B, Morphology of FUCCI lentiviral engineered cCICs expressing monomeric Kusabira Orange (mKO; G1 phase) and AzG (S/G2/M phases) fluorescence. BF, bright field. C‐E, Following neonatal (P3) intramyocardial injection, majority cCICs express both mKO and AzG at 7 dpi. Boxed area represented in (D, merged) and (E, mKO and AzG) (n = 3). F‐H, cCICs are still proliferative at 14 dpi indicated by AzG expression (green). Boxed area represented in (g, merged) and (h, mKO and AzG) (n = 3). I‐K, Majority of retained cCICs not proliferative at 21 dpi indicated mKO^+^ (red) AzG‐ expression (green). Boxed area represented in (J, merged) and (K, mKO and AzG) (n = 3). Scale bar, 50 μm

Extended persistence in the postnatal heart (Figure 3) led to experiments focused upon determining underlying mechanisms of cCIC retention and survival. Three distinct considerations were evaluated: (i) early retention after delivery, (ii) ongoing cell cycle activity of engrafted cCICs, and (iii) cCICs survival and host inflammatory response. First, early retention following delivery was assessed with injection of 5000 cCICs into a P3 heart. Percentages of cCICs retained in the neonatal heart at 2 and 48 hpi were 36.2% ± 17.0% (1812 ± 848) vs 33.4% ± 6.2% (1674 ± 535) as measured by enzymatic digestion followed by flow cytometry for mCherry^+^ cells (Figure S4a‐b). To contextualize the retention of cCICs in the neonate, comparative analysis was undertaken following established protocols from our group of 100 000 cells injected intramyocardially at the time of challenge into the infarct border zone of adult (P90) mice.^20^ In comparison, percentage of cCICs retained in the adult infarcted heart at 48 hpi was significantly lower at 5.2% ± 1.0% (5192 ± 954; *P* < .0001) (Figure S4c) verifying higher fractional initial cell retention in neonatal vs a pathologically injured adult heart. Second, cell cycle activity of cCICs retained in the postnatal heart was assessed using fluorescence ubiquitination‐based cell cycle indicator (FUCCI) labeling^35^, ^36^ (Figure 4A, see Section 4). FUCCI lentiviruses (cCIC^FUCCI^) carrying cell cycle indicators Geminin^+^ (Azami Green; AzG) and Cdt1^+^ (monomeric Kusabira Orange 2; mKO2) were used to transduce cCICs, and double‐positive cells were selected prior to intramyocardial injection by flow cytometric cell sorting (Figure 4B). cCICs^FUCCI^ cell cycle status was revealed by fluorescence of FUCCI indicators AzG and mKO. Engrafted cCIC^FUCCI^ exhibits both AzG and mKO2 fluorescence consistent with G1/S transition (AzG^+^/mKO2^+^) as well as G1 phase (AzG^−^/mKO2^+^) at 7 and 14 dpi (Figure 4c‐H). In comparison, by 21 dpi, the majority of cCICs are AzG^−^/mKO2^+^ with only a few AzG^+^/mKO2^+^ (Figure 4I‐K). Thus, cCICs delivered to the postnatal heart undergo cell cycle activity that diminishes between 14 and 21 dpi. Third, cCIC survival and host inflammatory response was evaluated by terminal deoxynucleotidyl transferase dUTP nick end labeling (TUNEL) assay and co‐immunostaining with the apoptotic marker cleaved caspase‐3 (CC‐3). Apoptotic activity was absent from cCICs negative for both TUNEL and cleaved caspase‐3 (Figure S5a‐b). Similarly, necrotic marker TNFα^+^ detected in injection site did not colocalize with remaining cCICs (Figure S5c). Inflammatory T lymphocytes (CD3^+^) infiltrates were undetectable at engrafted cCIC sites at 14 dpi (Figure S5d) but were found surrounding sparse cCICs at the peri‐epicardial region at 18 dpi (Figure S5e). Summing up findings related to persistence, initial retention is improved by cCICs delivery to postnatal hearts where cell cycle activity after engraftment is maintained and cell death avoided, although the maturing host immune response likely antagonizes persistence weeks after initial delivery.

### 2.6 Neonatal cardiac structural and functional development are not compromised by cCIC persistence

**Figure 5:**
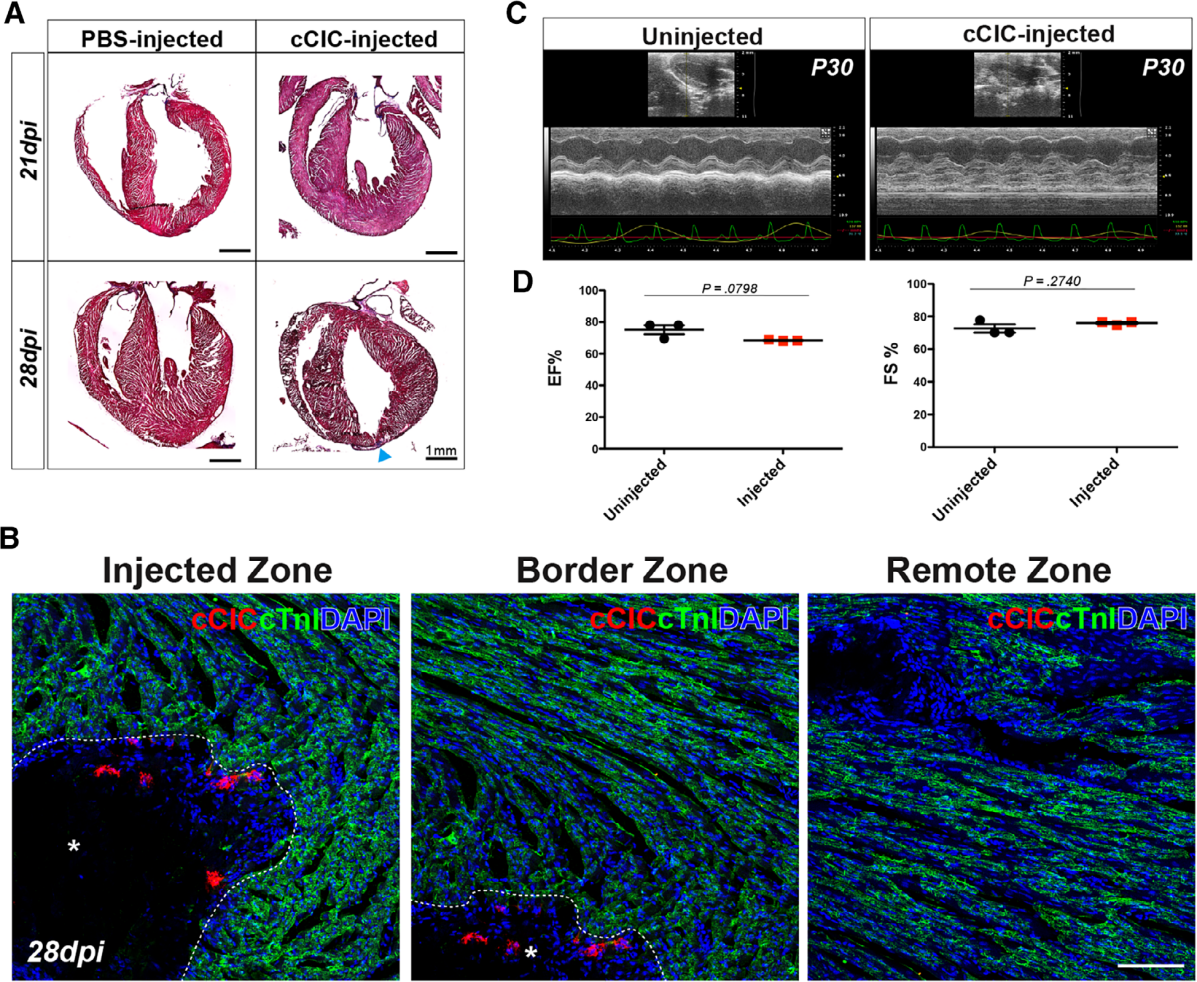
Neonatal cardiac structural and functional development is not compromised by c‐Kit^+^ cardiac interstitial cell (cCIC) persistence. A, Masson's Trichrome staining of phosphate‐buffered saline‐injected and cCIC‐injected hearts at 21 and 28 dpi. Small fibrotic area at 28 dpi in the left ventricular apex (arrowhead). B, Immunostaining of myocardium (cTnI) surrounding immediate injection zone (left, *), border zone (middle, *), and remote zone (right), showing structure of myocardium is morphologically normal at 28 dpi. C, Parasternal long‐axis echocardiography at P30, showing injected hearts are comparable to sham operated animals. Left: Sham, uninjected. Right: cCIC‐injected. D, Cardiac physiological functions are comparable between injected and uninjected animals. EF, ejection fraction. FS, fractional shortening. Unpaired Student's *t* test, two‐tailed (n = 3 hearts for each group). Scale bar, 100 μm

Extended engraftment and persistence of injected cCICs had minimal impact upon host myocardial structure and function assessed by histologic and echocardiographic analyses. Fibrotic remodeling in the region of injected cCICs was not markedly elevated from normal tissue at 21 dpi, with minimal deposition within the apical–pericardial region at 28 dpi by Masson's Trichrome staining (Figure 5A). cCIC‐injected hearts were structurally indistinguishable from phosphate‐buffered saline (PBS)‐injected control hearts, with gross morphology and myofibril arrangement at injection site, border zone, and remote zone comparable at 28 dpi by cardiac Troponin I immunolabeling (Figure 5B). Consistent with negligible impact of cCIC delivery upon myocardial structure, ejection fraction (EF) and fractional shortening (FS) were comparable between hearts receiving cCICs and uninjected age‐matched controls (Figure 5C,D). Collectively, these results demonstrate negligible impairment of myocardial structure or function consequential to cCIC persistence.

### 2.7 Polyploid DNA content of cCIC consistent with extra‐embryonic membrane localization following blastocyst injections

**Figure 6:**
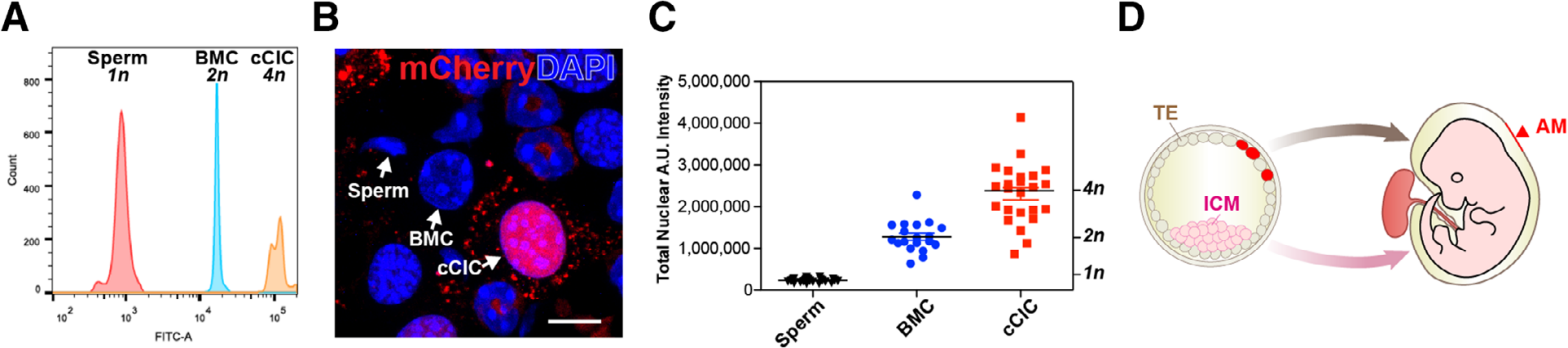
Polyploid DNA content of c‐Kit^+^ cardiac interstitial cell (cCIC) consistent with extra‐embryonic membrane localization following blastocyst injections. A, cCICs possess tetraploid (4n) DNA content relative to sperm (haploid, 1n) and bone marrow cell (BMC; diploid, 2n) as shown by flow cytometry. B and C, cCICs tetraploidy confirmed by confocal microscopy relative to BMC and sperm. Left, nuclear morphology. Right, quantitation of DAPI intensity (n = 26 for sperms, n = 19 for BMCs, n = 24 for cCICs). D, Cartoon model showing trophectoderm (TE)‐integrated cCICs (red) transitioning into patches in the AM (arrowhead), whereas ICM primarily gives rise to embryo proper (light pink). Scale bar, 10 μm

Developing embryos are comprised exclusively of diploid cells, whereas tetraploid cells are depleted from the epiblast lineage by mid‐gestation stage, excluded from the ICM, and instead reside among trophoblast layer contributing to extra‐embryonic membranes.^37^, ^38^ The extra‐embryonic membrane localization of blastocyst‐injected cCICs (Figure 1) is consistent with tetraploid DNA content of in vitro‐expanded cCIC.^23^ Tetraploid (4n) content of cCICs used for this study was confirmed by nuclear DNA content and larger nuclear size compared with sperm (haploid, 1n) or bone marrow cells (BMC, diploid, 2n) by flow cytometry and microscopy‐based nuclear intensity quantification (Figure 6A‐C). Thus, we posit that tetraploid exclusion during early embryonic development accounts for the extra‐embryonic membrane localization of cCIC blastocyst injections (Figure 6D), demonstrating phenotypic characteristics consistent with limited multipotentiality.

## 3 DISCUSSION

Biological activities of CIC continue to defy simple categorization, due in part to the heterogeneous nature of the population as well as inherent plasticity of individual cells.^39^, ^40^ CICs participate in all aspects of myocardial biology from development to maturation, homeostasis to aging, and acute injury to chronic remodeling.^1^, ^2^, ^3^ Regulatory functions of CICs in critical aspects of cardiac biology have spawned multiple approaches to influence their properties and activity with the goal of promoting beneficial action and mitigating maladaptive influences. After more than a decade of intensive investigation using various CICs expanded ex vivo to promote myocardial repair,^10^, ^15^, ^16^ much still remains unknown about adaptation of the cells, particularly with respect to culture conditions or reintroduction to intact myocardium. Even for the extensively characterized cCIC subpopulation, phenotypic properties and changes experienced by culture expanded cells upon reintroduction to a myocardial environment remain largely unknown. Heightened awareness of profound biological changes exerted by limited ex vivo culture expansion upon cCICs including transcriptional reprogramming^22^ and ploidy alteration^23^ emphasized the need to evaluate responsiveness of cCICs to myocardial exposure.

Marginal retention and subsequently poor survival of cCIC injected into adult myocardial tissue is a widely accepted limitation that hampers assessment of cellular biological activities occurring over several days to weeks. The strategy for overcoming this obstacle with ex vivo modifications to enhance cCIC engraftment and persistence with concomitant improvements in outcomes has been pursued by our group^20^, ^41^, ^42^ and others.^43^, ^44^, ^45^ However, such “unnatural” solutions to enhance cCIC engraftment and persistence deviate from widely used methodologies relying upon serial passaging of cells in standard culture conditions without manipulation of environmental conditions or molecular properties.^18^, ^19^, ^20^, ^45^ In the absence of interventions to enhance persistence, an alternative concept is to deliver cCIC to a myocardial environment possessing conditions that promote retention, growth, survival, and possibly integration. Following this alternative strategy, delivery of cCIC to cardiogenic fetal and neonatal environments should allow for prolonged presence and tracking to assess phenotypic adaptation. Precedents for this concept involving ESC chimeras^30^, ^31^, ^43^, ^46^, ^47^, ^48^ or fate‐mapping of cells introduced into cardiogenic environments^49^, ^50^ demonstrate that early developmental stages are particularly suited for assessing pluripotency and cellular plasticity. Thus, three distinct stages of embryonic, fetal, and neonatal development were used to interrogate phenotypic adaptation of cultured cCICs.

Embryogenesis is a spatiotemporally exquisite process. Rapid and dynamic cell migration, differentiation, and apoptosis occur at all time. At the blastocyst stage, a small number of blastomeres develop into the pluripotent ICM that gives rise to all three germ layers of the embryonic body for normal somatic and germ‐line contribution. The rest of the blastomeres differentiate into TE giving rise to extra‐embryonic tissues and supporting embryonic development.^30^, ^31^ Exclusion from the ICM (origin of future embryo proper) and integration into TE (origin of future amniotic membrane) demonstrates a novel facet of cCIC biology (Figure 6d). Similarly, chimeric placental tissue forms following injection of tetraploid hybrid cells into blastocysts.^51^ Cultured murine cCICs acquire tetraploid DNA content with serial passaging and override cellular senescence.^23^ Indeed, the tetraploid nature of cCICs (Figure 6) likely accounts for the mechanism behind engraftment into TE and amniotic membrane integration (Figure 1 and Figure 2F‐G), since embryo chimerism by blastocyst injection requires karyotypic normalcy of donor stem cells.^30^ Tetraploid exclusion from the embryo and polyploidy of extra‐embryonic membranes are fundamental biological properties of development.^37^, ^38^ The presence of c‐Kit^+^ cells in murine amniotic fluid and in the amnion^52^ presents a potential permissive milieu to host transplanted cCICs and a possible mechanism for amniotic membrane engraftment. Similar to findings reported here, extra‐embryonic membrane contribution for pluripotent human ES cells follows introduction into murine blastocysts.^53^ Intriguing commonality of cardioprotective action from infarction injury shared between cultured cCIC^18^, ^20^, ^42^, ^45^, ^54^ and trophoblast‐derived stem cells isolated from E3.5 blastocysts^55^ suggest additional biological similarities may exist between these cell types. Clearly, incorporation of cCIC into extra‐embryonic membranes following blastocyst injection demonstrates E3.5 to be a permissive environment for investigation of cCIC biological adaptation.

Unlike extra‐embryonic tissue integration observed in blastocysts, cCICs adopt fibroblast‐associated phenotypic traits in prenatal and neonatal hearts (Figures 2 and 3). Mixed engraftment in multiple sites including cardiac, noncardiac, and extra‐embryonic locations in the prenatal E15.5 environment demonstrates amniotic membrane is still permissive for cCIC engraftment. Furthermore, the developing fetus now tolerates the presence of tetraploid cCIC, but without preferential myocardial localization or expression of cardiogenic markers. Instead, persistent cCIC show Vim expression consistent with fibroblast phenotypic characteristics (Figure 2E). Clearly, donated cCIC lacks inherent multipotential capacity for direct contribution as tissue‐specific cell types within the host, presumably due to the loss of identity markers consequential to in vitro culture expansion.^22^

Efficient chimeric competency relies on pairing donor cell autonomous developmental timing with host organ developmental stages,^46^, ^47^, ^56^ a synchrony which is absent when donated cCIC are met with fetal or neonatal environments. Although cCIC fail to demonstrate multipotential commitment, the neonatal environment does allow for prolonged persistence. Following interaction between cCICs and the developing myocardial environment for weeks after delivery revealed several novel biological adaptations from both the donated cells as well as the host tissue.

The time course of 4 weeks from a postnatal to early adult heart yielded distinct features correlating concurrent myocardial maturation with cCIC adaptation. Although cell tracking and quantitation of persistence in situ can present methodological challenges, these issues were circumvented by following fluorescently tagged cells in frozen tissue sections to preserve native fluorescence and enable direct visualization without immunostaining. Furthermore, direct fluorescence visualization of FUCCI readouts allowed monitoring of cCIC cell cycle progression in host myocardium. From the outset when cCIC delivery occurs at the optimal P3 time point (Figure 3A), the reparative capacity of the postnatal heart that is present at P1‐P2 has largely been lost coinciding with cardiomyocyte exodus from cell cycle and increases in local ECM stiffness.^57^, ^58^, ^59^ Comparable phenotypic traits with cCIC previously found in the fetal context include expression of Vim (Figure 3J) but lack cardiac lineage markers (Figure 3B,C). Innate tissue reaction to the persistence of cCIC at 7 dpi is likely represented by accumulation of TenC, an ECM component associated with wound healing responses.^60^, ^61^, ^62^ The neonatal myocardium remains permissive for the exogenous cCICs, not only for initial retention (Figure S4) but also for ongoing cell cycle activity (Figure 4) and survival (Figure 5). Engrafted cCICs are well tolerated by the host myocardium up through 2 weeks after delivery (14 dpi), after which withdrawal from cell cycle progression, arrival of adaptive immune CD3^+^ T cells (Figure S5d‐e), and diminished morphologic features (Figure 3l‐m) heralds decline of the donated cCIC population. Persistence by cell fusion in neonatal injections is unlikely since cCICs often appear in large clusters (ranging from 100 to 500 μm), and numerous simultaneous cell fusion events all occurring at same location would be unprecedented. Cell death due to inflammation, apoptosis, or necrosis is a major cause for postinjection cell loss,^63^ but scant evidence of these processes in donated cCIC (Figure S5) is consistent with their prolonged persistence in the postnatal heart.

Persistence of injected cCIC in neonatal hearts for up to 4 weeks (28 dpi) is remarkable given long‐standing issues of retention and engraftment in the adult heart. Donated cells are typically lost shortly after delivery with engraftment rates below 5% to 10% by 24 hpi and less than 2% by 48 hpi.^24^, ^25^, ^26^, ^27^ In comparison, initial cCIC engraftment of 36.2% ± 17.0% at 2 hpi remained high at 33.4% ± 6.2% by 48 hpi in neonatal injections (Figure S4). Moreover, histological analyses at the 4‐week termination point for the study showed foci of remaining cCICs without fibrotic remodeling, preservation of local cardiomyocyte myofibrillar organization, and negligible impact upon myocardial structure (Figure 5A,B). Cardiac function in juvenile mice that matured with engrafted myocardial cCIC possesses contractile function indistinguishable from uninjected normal control mice at 1 month of age (Figure 5c‐d). The prevailing theory for mechanism of action in cell therapy involves paracrine effects including secretion of protective molecules and activation of endogenous reparative processes^64^, ^65^, ^66^ facilitated by higher retention and persistence of injected cells in the neonatal heart consistent with our results. However, the human heart requires years to fully mature and specific developmental stages and mechanisms for optimal donor cell retention remain to be determined.

Looking ahead, conclusions from this study confirm the influence of microenvironments upon cell fate as well as limited multipotentiality remaining a consideration when using ex vivo expanded adult‐derived stem cells. Developing hearts and blastocysts are permissive environments for prolonged persistence of cCIC, with differential fate outcomes influenced by host tissues. cCIC fate was directed toward fibroblast or extra‐embryonic membrane phenotypes. Neonatal hearts developing into adolescence with persistent cCICs were comparable to normal uninjected hearts in terms of myocardial maturation, structure, and contractile performance. Our study represents (to our knowledge) the first demonstration of significant cCIC retention and persistence in a natural damage‐free environment. The neonatal heart can therefore serve as an in vivo platform for future studies intended to assess cCIC biological activity and the spatiotemporal dynamics of host myocardium undergoing development and remodeling with exogenously introduced cells.

## 4 MATERIALS AND METHODS

All animal protocols and studies were approved by the review board of the Institutional Animal Care and Use Committee at San Diego State University.

### 4.1 Mouse cCIC isolation and fluorescence engineering

CICs were isolated from 8‐week‐old FVB/J mice by enzymatic dissociation (Collagenase II, 460 U/mL, Worthington, LS004174) of the whole heart on a Langendorff apparatus (Radnoti, 158831) as previously described.^20^ Following myocyte depletion, Lin^−^CD45^−^c‐Kit^+^ cCICs were obtained by removing lineage^+^ and CD45^+^ fraction using lineage depletion Kit (Miltenyi, 130‐110‐470) and CD45 MicroBeads (Miltenyi, 130‐052‐301), followed by c‐Kit^+^ cCICs enrichment (Miltenyi, 130‐091‐224) by magnetic activated cell sorting. Cells were expanded in growth media [DMEM/F12 (Gibco, 11330032) supplemented with 10% ES‐FBS (Gibco, 16141079), 10 ng/mL basic fibroblast growth factor (FGF; BioPioneer, HRP‐0011), 20 ng/mL EGF (Sigma‐Aldrich, E9644), 1× ITS (Lonza, 17‐838Z), 10 ng/mL LIF (BioPioneer, SC‐041‐2), and 1X (Gibco, 10378016)] and passaged every 2‐3 days to maintain at a confluence of ≤40%. Cultured cCICs were transduced with lentiviral PGK‐mCherry construct at MOI of five and puromycin selected to stably express mCherry fluorescence. cCICs used in mCherry experiments were isolated from two male mice, and cCICs used in FUCCI experiments were isolated from four mice (2 males + 2 females).

### 4.2 cCIC‐sphere formation

For cell aggregation, 2.75 × 10^6^ cCICs were plated in 5 mL EB medium (KnockOut DMEM [Gibco 10829‐018] supplemented with 15% KnockOut Serum Replacement [Gibco 10828‐028], 0.1 mM MEM Non‐Essential Amino Acids Solution [Gibco 11140‐050], 1X GlutaMAX‐I [Gibco 35050‐079]) in low‐attachment petri dish for 4 days at 37°C, 5% CO_2_. For mesoderm induction, cCIC‐spheres were transferred to AF‐coated tissue culture dish in EB medium supplemented with 10% ES‐FBS to allow attachment overnight, followed by mesodermal induction media ([Gibco, 31980030] and Ham's F12 [HyClone, SH30026.01] supplemented with 5 ng/mL Activin A [Peprotech, 120‐14E], 0.5 ng/mL BMP4 [Peprotech, 120‐05ET], 5 ng/mL human vascular endothelial growth factor—VEGF [Peprotech, 100‐20], and 1X Pen/Strep [Gibco, 15140163]) for 24 hours, cardiac induction media (StemPro‐34 SFM medium [Gibco, 10639011] supplemented with 2 mM l‐glutamine [Gibco, 25030081], 0.5 mM Ascorbic acid [Sigma‐Aldrich, A4403‐100MG], 5 ng/mL human VEGF, 10 ng/mL human basic FGF, and 50 ng/mL human FGF10 [Peprotech, 100‐26]) for 7 days. Subsequently, cells were washed twice in cold PBS and fixed in 1% paraformaldehyde (PFA) for immunocytochemistry. For protein lysates, cell pellets were collected before mesodermal induction and at the end of cardiac induction.

### 4.3 Histology and immunofluorescence staining

Mice were heparinized (Sigma‐Aldrich H3393, 10 units/g) by intraperitoneal injection and euthanized at harvest time points. For animals younger than 14 days, euthanasia was carried out by anesthetization on ice followed by decapitation. For animals at 14 days and older, euthanasia was carried out by isoflurane overdose followed by cervical dislocation. Hearts were perfused with PBS and 1% PFA before removal from thoracic cavity, followed by fixation in 1% PFA immersion overnight at 4°C. Fixed hearts were dehydrated in 30% sucrose in PBS overnight at 4°C, then in OCT + 30% Sucrose mix at 1:1 ratio, before mounting in NEG50 and frozen on dry ice. Frozen sections were cut at 20 μm thickness and collected onto Superfrost glass slides. Sections were allowed to dry for 48 hours prior to storage at −20°C.

Following equilibrium at RT for 5 minutes and brief rehydration in PBS, frozen tissue sections were incubated in permeabilization solution (0.1% Triton X‐100, 0.1 M glycine, 1% bovine serum albumin [BSA] in PBS) for 30 minutes at room temperature (RT), then blocked in blocking solution (10% donkey serum [Millipore, S30‐100 mL], 0.1 M glycine, 1% BSA in PBS) for 1 hour at RT. Cells grown and fixed in chamber slides were permeabilized for 15 minutes and blocked for 1 hour prior to antibody staining. Following blocking, samples were incubated overnight in primary antibodies at 4°C (see dilutions in Table S1), washed in PBS, and incubated in secondary antibodies (1:100) for 90 minutes at RT. All samples were counterstained with DAPI (Sigma‐Aldrich D9542, 0.1 μg/mL) and mounted in VectaShield and imaged by Leica SP8 confocal microscopy.

### 4.4 Immunoblotting

At the time of harvesting, cells were washed twice in cold PBS and lysed in RIPA buffer (Thermo, 89901) with freshly added proteinase inhibitor and phosphatase inhibitors cocktails (Sigma P0044, P8340, P5726) for 30 minutes on ice with intermittent vortexing. Cell lysates were then centrifuged for 10 minutes at 11000*g* at 4°C to remove insoluble debris. Supernatants were quantified with Bradford assay (ThermoFisher, 23236) and 20 μg lysates were run on 4% to 12% Bis‐Tris protein gels (Invitrogen, NP0335BOX) and transferred onto a PVDF membrane (Millipore, IPFL00010), followed by blocking in 10% nonfat dry milk (LabScientific) for 1 hour at RT. Primary antibodies (see dilutions in Table S1) were incubated overnight at 4°C and secondary antibodies (1:1000) for 90 minutes at RT. Immunoblots were scanned with LI‐COR Odyssey Clx system.

### 4.5 Quantitative RT‐PCR

Total RNA was isolated using Quick‐RNA MiniPrep kit (Zymo Research, R1055) following manufacturer's protocol. RNA concentration was determined using NanoDrop 2000 spectrophotometer (ThermoFisher) and normalized to 500 ng for cDNA synthesis by iScript cDNA synthesis kit (BioRad, 170‐8891). Of note, 6.5 ng of cDNA was used for each qPCR reaction using iQ SYBER Green (BioRad, 170‐8882) on a CFX Real‐Time PCR thermocycler (BioRad). Primers and sequences used in this study are listed in Table S2. Ct values were normalized to *Actb* and analyzed by the ∆∆Ct method relative to ESCs.

### 4.6 Generation of mouse chimera: Blastocyst isolation, injection, and uterine transfer

Superovulated FVB/J females at 4‐5 weeks of age were mated with FVB/J males overnight. The next morning, mating was confirmed by vaginal plug, and mated females (0.5 days postcoitum, dpc) were euthanized by cervical dislocation for collection of zygotes from oviduct. Zona pellucida was removed by brief digestion in hyaluronidase. Alternatively, 3.5 dpc females were euthanized and uterine horns were flushed with M2 media (Millipore, MR‐015‐D) for collection of morula. Zygote and morula were both collected in M2 and cultured in preequilibrated KSOM media bubbles (Millipore, MR‐106‐D) under mineral oil immersion (Sigma, M8410) at 37°C (5% CO_2_, humidified) until blastocyst injection.

For blastocyst injection, cultured cCICs were trypsinized and pelleted in growth media supplemented with 1× HEPES (Gibco, 15 630 080). Approximately 8 to 12 cells were injected into each blastocyst. Following injection, blastocysts were washed in M2 and allowed to recover in KSOM for 30 minutes before uterine transfer. Approximately 15 to 20 blastocysts were transferred into the uterus of 2.5 dpc pseudopregnant recipient B6/CBA females mated with vasectomized Swiss Webster males. Alternatively, 20 to 25 blastocysts were transferred into the uterus of 0.5 dpc pseudopregnant B6/CBA females. FVB/J background GFP^+^ESCs were used as chimera generation control.

### 4.7 Whole‐mount blastocyst immunostaining and 3D reconstruction

CIC‐injected blastocysts were incubated in preequilibrated KSOM media for 48 hours at 37°C (5% CO_2_, humidified). Postinjection blastocysts at 24 hpi and 48 hpi were fixed in 1% PFA overnight at 4°C. Blastocysts were washed in PBST (PBS + 0.1% Tween‐20), incubated in 0.1% Triton X‐100, 1% BSA, 0.1 M glycine, and 10% donkey serum in PBST for 30 minutes at RT. Primary antibodies (see dilutions in Table S1) were incubated overnight at 4°C, and secondary antibodies (1:100) were incubated for 1.5 hours at RT. DAPI was added to last PBST washes to stain nuclei. All washes and incubations were performed in liquid bubbles under mineral oil immersion. Following staining, blastocysts were gradually transferred from PBST to 20%, 50%, and 70% glycerol, and mounted in 80% glycerol. Z‐stack series scanning was performed using Leica SP8 confocal microscopy (×63) at a 5‐μm interval depth. Three‐dimensional reconstruction videos were generated using Leica LAS X analysis software.

### 4.8 In utero transplantation

Timed pregnant FVB/J female inbred mice were anesthetized with ketamine/xylazine according to body weight at 10 μL/g. Uterine horns were exteriorized through a short ventral midline incision at lower abdomen. Cells were delivered using a microcapillary needle with the appropriate volume of cell suspension at approximately 5000 cells per embryo into pericardial space. After injection, the uterine horns were gently placed back into the abdomen, and the maternal abdominal muscle and peritoneum were closed by surgical adhesive. Following recovery, two buprenorphine doses (0.2 μg/body weight g) were given every 12 hours as analgesia. At 2 dpi, dams were euthanized by isoflurane overdose followed by cervical dislocation. Embryos were dissected out of uteri in cold PBS and fixed in 1% PFA immersion at 4°C overnight.

### 4.9 FUCCI constructs and expression

The FUCCI system consists of two chimeric proteins, mKO‐Cdt1 and AzG‐Geminin, which oscillate reciprocally during cell cycle, labeling the nuclei in G1 phase orange and those in S/G2/M phases green.^35^ During G1/S transition, both probes are present, resulting in a yellow fluorescence (overlaid green and red); during the brief gap between M and G1 phases, neither probe is present and fluorescence is absent. Oscillation between red, yellow, and green signals tracks cell cycle status^35^, ^36^ (Figure 4A). FUCCI lentiviral plasmids were generated as previously described.^36^ For FUCCI expression, cCICs were transduced with lentiviral PGK‐Cdt1‐mKO and PGK‐Gem‐AzG constructs at MOI of 2.5 of each construct and sorted for mKO^+^/AzG^+^ double positivity by flow cytometry (BD, Canto).

### 4.10 Postnatal intramyocardial cell delivery

FVB/J neonates were anesthetized by hypothermia on ice for 1 to 3 minutes until immobile. Anesthesia was maintained by placing pups on an ice‐filled petri dish throughout the procedure. Peristernal thoracotomy was performed by making a small incision at the fourth intercostal space. Intercostal muscles were separated by blunt lateral dissection in order to facilitate access to the heart. After expanding the fourth intercostal space, the apex was gently stabilized using curved forceps. With gentle pressure on the abdomen, hearts can be exteriorized and stabilized with microforceps without damaging myocardium. Cells were delivered via a flame‐pulled glass capillary needle (opening diameter ~50 μm, calibrated by hemocytometer) with tangential angle into the myocardium, and titrated volume was injected by mouth pipetting (Sigma, A5177). Approximately 5000‐10 000 cells were delivered in a total of 2.5 μL via three injection sites tangential to the LV apex region. After injection, the heart was returned to thoracic cavity, and muscle and skin incision was closed using surgical adhesive (Meridian, Surgi‐lock 2oc). Postinjection pups were warmed up rapidly on a heating pad for several minutes until recovery (body color turns pink and spontaneous movement), followed by mixing the pups with dam's bedding in order to reduce the chances of cannibalization. Postop pups were returned to the dam and littermates as soon as possible and maternal acceptance was monitored. The whole surgical procedure should be completed within 10 minutes to minimize the time spent separated from the mother and to improve survival. At 7, 14, 21, and 28 dpi, injected hearts were collected and washed twice in cold PBS, followed by fixation in 1% PFA at 4°C overnight.

### 4.11 Myocardial infarction and intramyocardial injection

Myocardial infarction and intramyocardial injection were carried out as previously described^67^ on FVB/J strain mice. Briefly, hearts were popped out through the fourth intercostal space and the left anterior descending artery (LAD) was permanently ligated at the second distal branching point using a 7‐0 silk suture. Following LAD ligation, three injections were delivered (Harvard Apparatus, Hamilton infusion pump) at the border zone surrounding the blanching area at a tangential angle parallel to the myocardial wall, in order to ensure intramyocardial cell delivery. A total of 100 000 cells/10 μL were injected per heart at the three injection sites. Following injection, the heart was immediately placed back into the intrathoracic space and muscle and skin were closed by surgical adhesive.

### 4.12 Cardiac cell disassembly and quantification

Postinjection hearts were enzymatically disassembled into single‐cell suspension and subjected to flow cytometry for fluorescence‐based cell count. For neonates, postop pups at 2 and 48 hpi were heparinized and anesthetized on ice. Anesthesia was maintained by hypothermia in a petri dish filled with ice during the surgical procedure. Perfusion and digestion were performed following a modified protocol as previously described.^68^ Briefly, the heart was digested (Collagenase II, 460 U/mL) by continuous perfusion through the LV apex with the aortic arch clamped (5 minutes at 1 mL/minute). The digested tissue was then triturated and transferred into a 15‐mL conical tube for subsequent digestion for 15 to 30 minutes in a 37°C water bath with agitation. All cell suspensions were filtered through a 75‐μm cell strainer to exclude cardiomyocytes and tissue debris. The flow‐through was pelleted by centrifugation at 350*g* for 10 minutes. Cell pellets were then resuspended in 500 μL PBS/0.5% BSA and subjected to flow cytometry count.

For quantitative analysis from adult heart injection, cardiomyocytes must be removed due to their rod‐shape and large cell size exceeding the capacity of the flow cytometer. Only the non‐myocyte population was used for cell count. Non‐myocytes were obtained from post‐myocardial infarction hearts at 48 hpi. As described in the cCIC isolation method, postop hearts were enzymatically digested (Collagenase II, 460 U/mL) on a Langendorff apparatus (12‐18 minutes at 1 mL/minute), triturated, and filtered through a 100‐μm cell strainer to remove undigested debris. The supernatant was then sequentially filtered through 40‐ and 30‐μm cell strainers. The flow‐through containing all non‐myocytes was pelleted by centrifugation at 350*g* for 10 minutes. Cell pellets were then resuspended in 1 mL PBS/0.5% BSA and subjected to flow cytometry count.

### 4.13 Flow cytometry

Single‐cell resuspension was analyzed using a BD FACSCanto instrument. Cells digested from sham (uninjected) hearts were used to exclude autofluorescence disturbance, and cultured cCICs expressing mCherry fluorescence were used as positive gating to establish fluorescence levels. All cells from neonatal hearts were analyzed. A recorded volume of 100 to 200 μL cell suspension from adult interstitial cells was analyzed, and the whole heart cell count was calculated based on volumetric ratio relative to 1 mL initial cell suspension. Flow cytometry data were analyzed by FlowJo software (BD Biosciences).

### 4.14 Echocardiography

Echocardiography was performed using the Vevo2100 (Visual Sonics) system from LV parasternal long and short axes at a heart rate range of 500‐550 beats/minute. EF and FS were determined by offline analysis. Age‐matching unoperated mice were used as baseline controls.

### 4.15 Masson's trichrome staining

Masson's trichrome staining was performed using trichrome stain kits following the manufacturer's protocol (Sigma‐Aldrich, HT15). Briefly, frozen tissue sections were rehydrated in PBS for 5 minutes and post‐fixed in 10% formalin for 1 hour at RT, followed by fixation in Bouin's solution overnight at RT. The next day, sections were washed in water and subjected to a series of staining in Weigert's Iron Hematoxylin Solution for 5 minutes, Biebrich Scarlet‐Acid Fuchsin for 5 minutes, Phosphotungstic/Phosphomolybdic Acid Solution for 5 minutes, Aniline Blue Solution for 5 minutes, and 1% acetic acid for 2 minutes with washes in deionized water in between. Finally, sections were gradually dehydrated through alcohol and cleared in xylene for 3 minutes before mounting in Permanox. All images were scanned by the Leica DMIL600 microscope using the xy stage tilescan and automatically stitched by the Leica LAS X analysis software.

### 4.16 Cell death detection

TUNEL assay was performed using an in situ cell death detection kit (Roche 11684795910) following the manufacturer's protocol. Briefly, frozen tissue sections were rehydrated in PBS for 5 minutes at RT, postfixed in 4% PFA in PBS for 20 minutes, and permeabilized in 0.1% Triton X‐100, 0.1% sodium citrate for 2 minutes at 4°C. Following brief wash in PBS, samples were incubated in the TUNEL reaction mixture (Label solution + Enzyme solution, 9:1) for 1 hour at 37°C. Samples were then washed in PBS, mounted in VectaShield, and scanned using a Leica SP8 confocal microscope.

### 4.17 Ploidy quantification

Following euthanization, mouse sperm was collected from vas deferens and maintained in PBS/0.5% BSA on ice. BMC were collected from femur flushed with PBS/0.5% BSA using a 27‐gauge needle and filtered through a 30‐μm cell strainer to remove debris. Cultured cCICs were trypsinized and pelleted at 300*g* for 5 minutes. Cells were then stained with Sytox Green (Invitrogen, S7020, 1 μM) for 15 minutes at RT before subjected to flow cytometry analysis. Unstained cells of each cell type served as negative gating controls. Ploidy comparison was established using sperm as haploid and BMC as diploid control using FlowJo software.

Alternatively, sperm, BMC, and cCIC suspensions were manually mixed and cytospun (Thermo, Cytospin 4) for 3 minutes at 800 rpm with low acceleration onto a poly‐d‐lysine‐coated slide. Cells were then fixed in 1% PFA for 20 minutes at RT, stained with DAPI for 5 minutes at RT, following by three PBS washes to remove excess staining. cCIC nuclei were identified by mCherry fluorescence, BMC nuclei were identified by mCherry negativity, and sperm nuclei were identified by unique fishhook‐like nuclear morphology. Nuclear DAPI signals were scanned by z‐series spanning entire nucleus at 1 μm interval using Leica SP8 confocal microscopy. Z‐projection was reconstructed with sum intensity by ImageJ. Nuclear intensity was quantified by nuclear volume tracing using ImageJ and presented as arbitrary units.

### 4.18 Statistical analysis

All data were presented as mean ± SEM and analyzed by GraphPad Prism 5.0b with unpaired Student's *t*‐test, two‐tailed. A *P*‐value <.05 was considered statistically significant.

## CONFLICT OF INTEREST

M.A.S. is a founding member of CardioCreate, Inc. The remaining authors declared no potential conflicts of interest.

## AUTHOR CONTRIBUTIONS

B.J.W.: designed the overall experiments, performed the experiments, analyzed the data, wrote the manuscript; R.A.: designed the overall experiments, performed the experiments; M.A.S.: designed the overall experiments, wrote the manuscript; A.M., S.S., J.W.: performed the experiments; R.S., M.M.: performed the experiments, and analyzed the data; all authors read and approved the final manuscript.

## Supporting information


**Appendix S1.** Supporting Information

**Video S1**

**Video S2**

## Data Availability

The data that support the findings of this study are available from the corresponding author upon reasonable request
