# Supplementary material for: Adaptation within embryonic and neonatal heart environment reveals alternative fates for adult c‐kit+ cardiac interstitial cells
Source: Stem Cells Transl Med. 2019 Dec 31;9(5):620–35. doi: 10.1002/sctm.19-0277 (PMC7180292; doi:10.1002/sctm.19-0277)
Supplement: Supplementary file 1 — Appendix S1. Supporting Information [file SCT3-9-620-s001.pdf]

Supplementary Materials

Figure S1

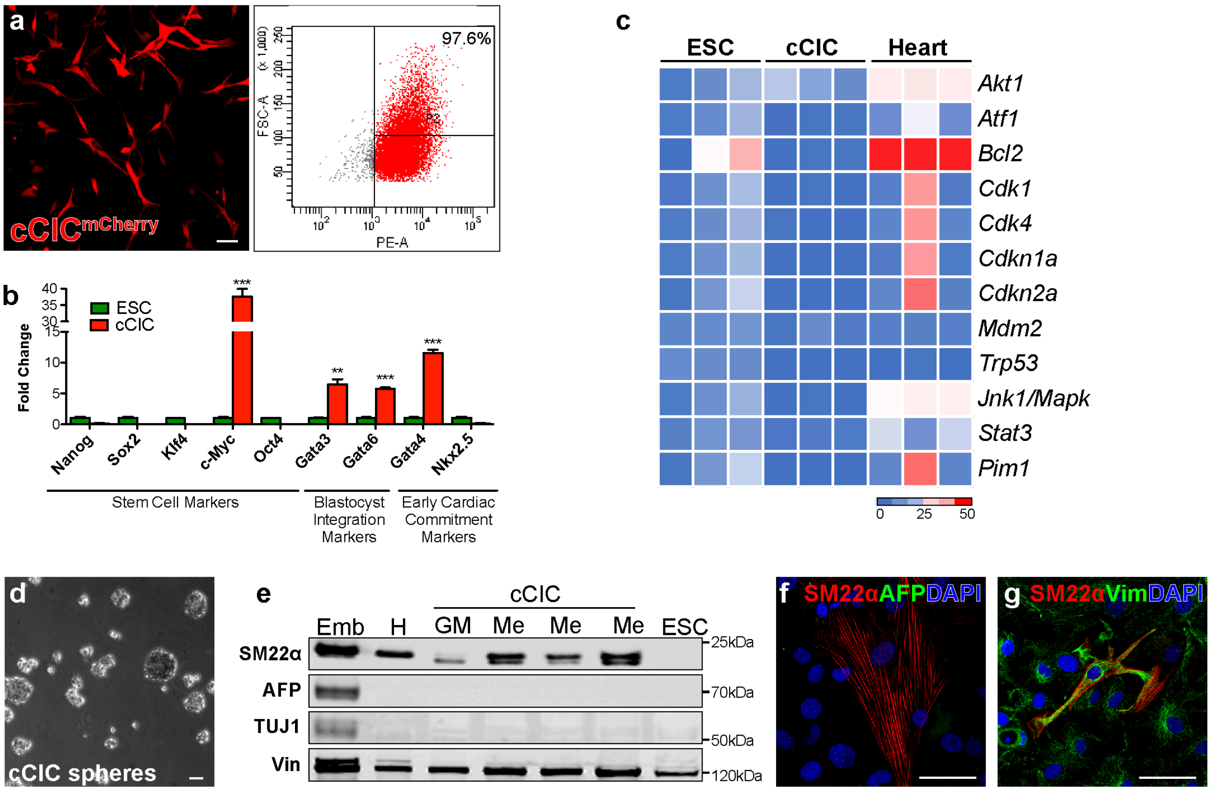

Figure S1. Mesodermal potential maintained by cCIC *in vitro.*

(a) Morphology of cCICs isolated from adult heart with lentiviral engineered mCherry fluorescence, flow cytometry plot shows 97.6% cells are mCherry^{+}. (b) Gene expression comparison of stem cell markers, blastocyst integration markers, and early cardiac commitment markers between cCICs and ESCs by qRT-PCR. Mean ± SEM, $^{**}P < 0.01$, $^{***}P < 0.0001$ vs. ESC, n=4. Unpaired student t test, two-tailed. (c) Heatmap of pro-oncogene expressions of ESC, cCIC, and whole heart by qRT-PCR. Color scale represents fold change relative to ESC. n = 3. (d) Morphology of spheres formed by cCICs at day 4 (n = 4). (e) Immunoblotting showing cCICs display mesodermal potential after differentiation for 7 days. Emb: E10.5 whole embryo lysate. H: P30 adult heart. GM: growth media, undifferentiated. Me: Mesoderm induction, differentiated. SM22α: Smooth muscle 22α, mesoderm. AFP: α-Fetoprotein, endoderm. TUJ1: βIII Tubulin, ectoderm. Vin: Vinculin, loading control. (f) Immunostaining showing cCICs are positive for SM22α and negative of AFP expression after 7-day mesodermal induction. (g) Immunostaining showing majority of cCICs express Vim (Vimentin), fibroblast marker. Scale bar, 50μm.

Figure S2

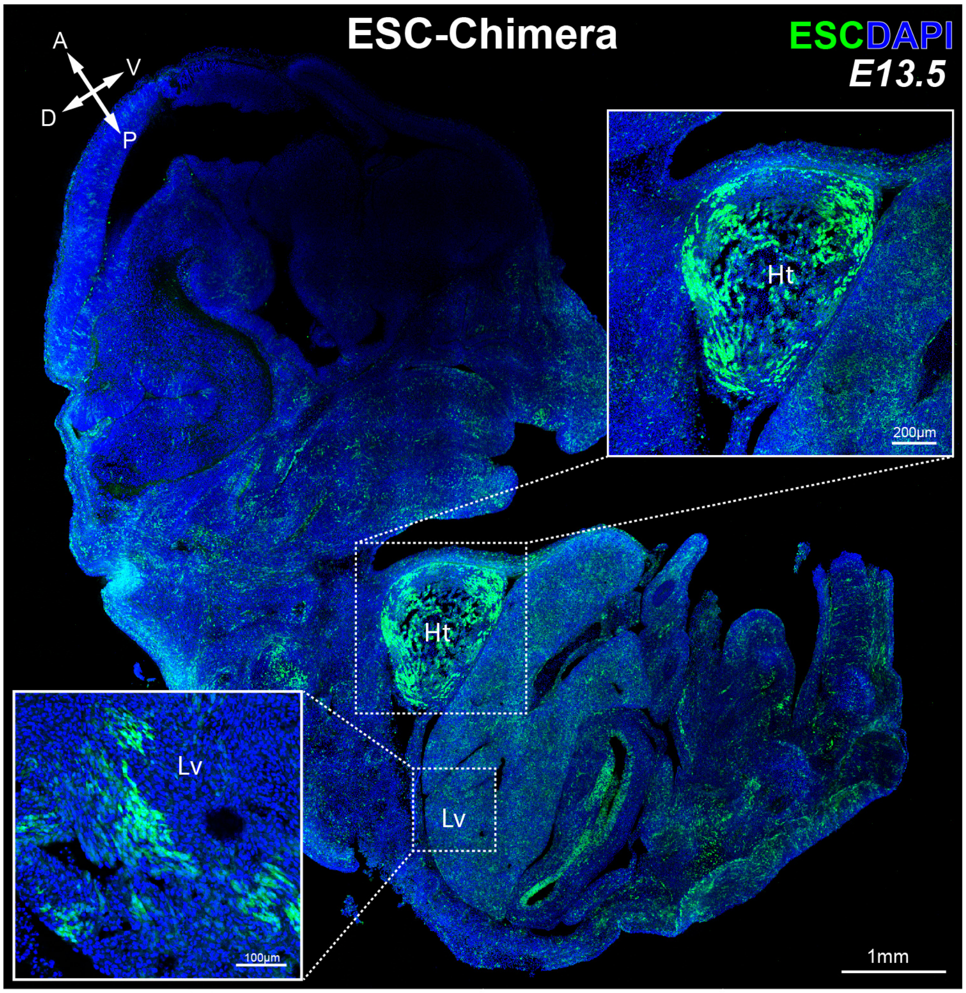

Figure S2. Chimera generation by ESCs.
E13.5 ESC-Chimera with mosaic ESC-GFP integration pattern. Insets: high magnification of indicated organs. H: heart. Lv: Liver. Cross arrows: anatomical plane. A: anterior, P: posterior, D: dorsal, V: ventral. n = 10/52. Scale bar, 1mm.

Figure S3

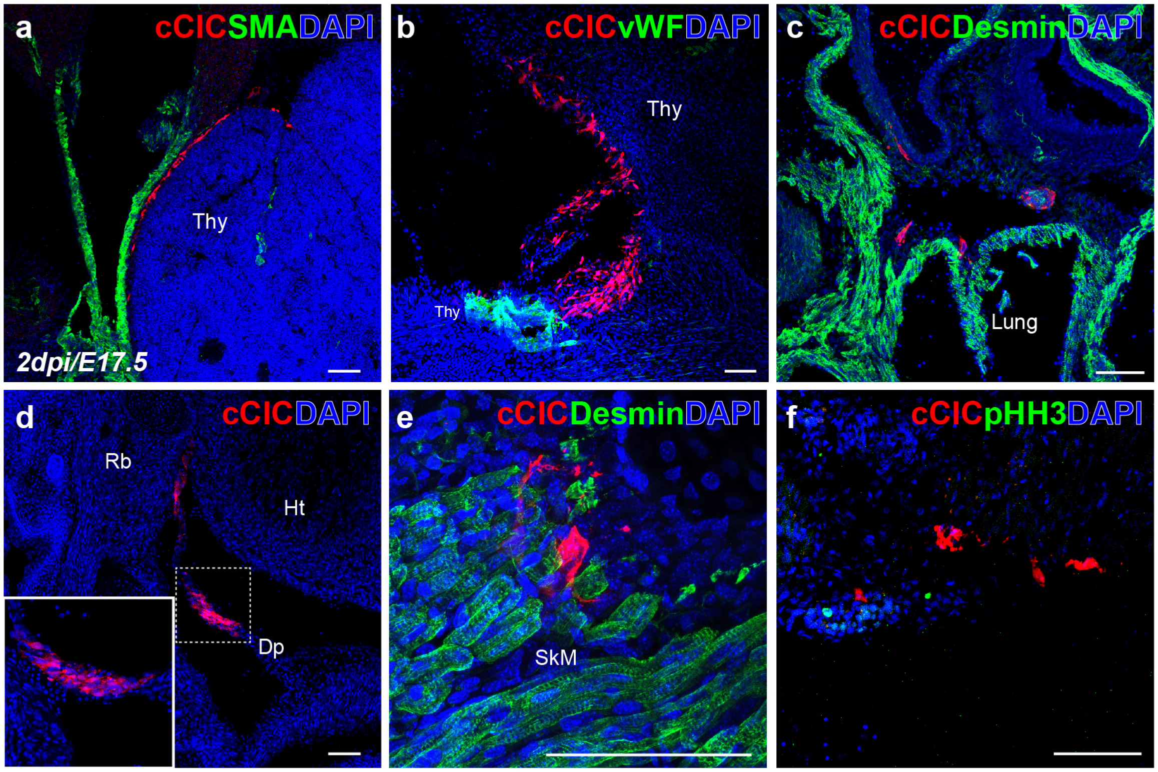

Figure S3. IUT delivered cCICs are detected in extracardiac tissues.
(a, b) cCICs were found along Thymus. Thy, thymus. (c) cCICs distributed within lung. (d) cCICs integrated into and aligned within diaphragm. (e) cCICs integrated among skeletal muscle. Dp, diaphragm. Ht, heart. Rb, ribs. SkM, skeletal muscle. (f) Immunostaining of proliferation marker Phospho-Histone H3 (pHH3, green) showing cCICs remaining in vicinity of the heart are not in M phase at 2dpi. n = 4/6. Scale bar, 100μm.

Figure S4

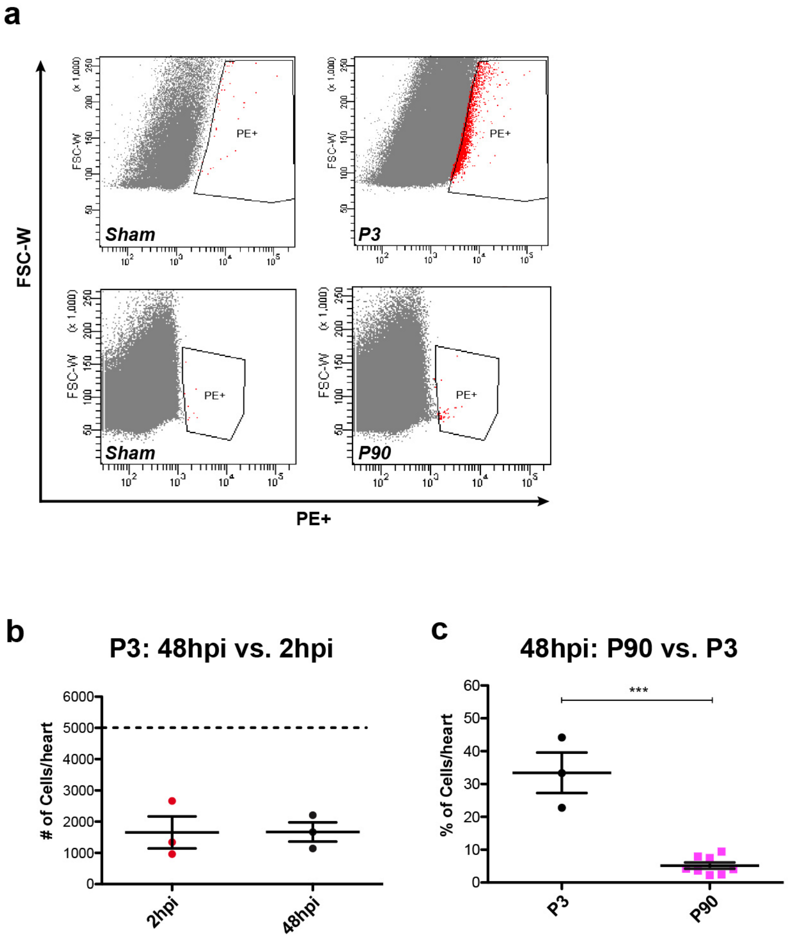

Figure S4. Comparison of cell retention between neonatal and adult heart recipient. (a) Representative flow cytometry dot plots of cCIC counts from P3 and P90 injections. Top: P3 injections at 2hpi. Bottom: P90 injections at 48hpi. (b) Number of injected cCICs detected at 2hpi and 48hpi per heart at P3. Dashed line: 5 000 cells injected per heart. Unpaired student t test, two-tailed (n = 3). (c) Percentage of injected cCICs detected at 48hpi per heart. P3: 5 000 cells injected per heart. P90: 100 000 cells injected per heart. Mean ± SEM, ***P <0.0001. Unpaired student t test, two-tailed (n = 3-8 hearts for each group).

Figure S5

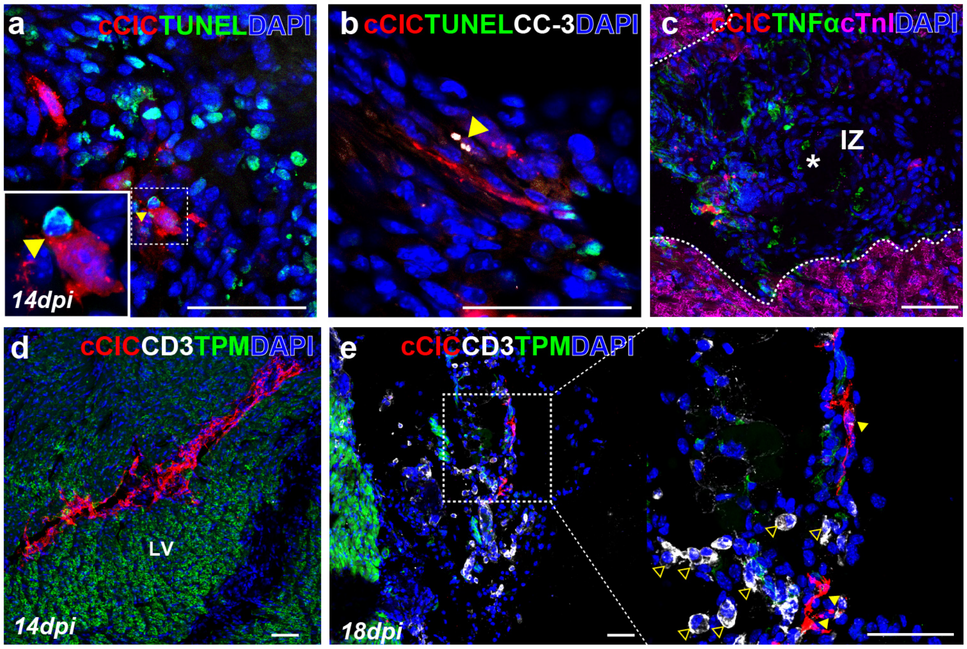

Figure S5. cCICs long term survival and host inflammatory response.
(a) TUNEL staining showing cCICs do not express TUNEL (green) at 14dpi. Inset: high magnification of a cCIC adjacent to a TUNEL positive cell (arrowhead). (b) TUNEL (green) and Cleaved-Caspase 3 (CC3, white, arrowhead) immunostaining showing cCICs are negative of both apoptotic markers at 14dpi. n = 3. (c) cCICs do not colocalize with TNFα (green) in IZ (*). IZ, injection zone. (d) Immunostaining of CD3 showing no infiltrating T cells around engrafted cCICs at 14dpi. (e) A cluster of $CD3^+$ T cells (arrowhead, clear) was detected surrounding cCICs (arrowhead, yellow) at peripheral region of epicardium at 18dpi. n = 3. Scale bar, 50μm.

Supplementary Online Video Legends

Supplementary online Video 1. Z-series of CICs ICM integration
Z-stack series of CICs retained in ICM and blastocoel by 24hpi. Z-size 100μm at 10μm interval. Red, native fluorescent of CICs. BF, bright field of blastocyst.

Supplementary online Video 2. 3D reconstruction of CICs anchoring in blastocyst
Three-dimensional reconstruction of whole-mount immunostaining at 48hpi showing CICs anchored with host cells and spread out as spindle morphology in a hatching blastocyst. Red, native fluorescence of CICs, unstained. Green, CDX2 trophectoderm. White, ZO1 tight junction. Blue, DAPI.

Supplementary Tables

Table S1. List of Antibodies

Name	Vendor/Catalogue	Dilution
AFP	R&D AF5369	1:100, 1:400 (IB)
CD3	Abcam, ab11089	1:100
CDX2	Abcam, ab157524	1:100
Cleaved Caspsase-3	Cell Signaling, 9661	1:100
cTnI	Abcam, ab56357	1:100
Desmin	Abcam, ab15200	1:100
HNA	Abcam, ab191181	1:100
Laminin	Abcam, ab11575	1:100
mCherry	ThermoFisher, M11217	1:200
Oct3/4	Santa Cruz Biotech, sc-5279	1:25
pHH3 (S10)	Abcam, ab47297	1:100
SM22α	Abcam, ab14106	1:100, 1:500 (IB)
SMA	Sigma Aldrich, A5228	1:100
TenC	RND, MAB2138	1:100
TNFα	Santa Cruz Biotech, sc-52746	1:100
TPM	Sigma Aldrich, T2780	1:100
TUJ	Sigma, T8660	1:400 (IB)
Vimentin	ThermoFisher, PA1-16759	1:200
Vinculin	Sigma, V9131	1:1 000 (IB)
vWF	Dako, A0082	1:200
ZO1	ThermoFisher, 617300	1:50

Table S2. List of Primers

Gene	Forward 5'-3'	Reverse 5'-3'
Actb	CTCTGGCTCCTAGCACCATGAAGA	GTAAAACGCAGCTCAGTAACAGTCCG
Akt1	ATGAACGACGTAGCCATTGTG	TTGTAGCCAATAAAGGTGCCAT
Atf1	GATTCCCACAAGAGTAACACGAC	CCTATGCTGTCAGATGAGTCCT
Bcl2	ATGCCTTTGTGGAACTATATGGC	GGTATGCACCCAGAGTGATGC
c-Myc	ACCACCAGCAGCGACTCTGAAG	GGGTGCGGCGTAGTTGTGCT
Cdk1	AGAAGGTACTTACGGTGTGGT	GAGAGATTTCCCGAATTGCAGT
Cdk4	ATGGCTGCCACTCGATATGAA	TCCTCCATTAGGAACTCTCACAC
Cdkn1a	CCTGGTGATGTCCGACCTG	CCATGAGCGCATCGCAATC
Cdkn2a	CGCAGGTTCTTGGTCACTGT	TGTTCACGAAAGCCAGAGCG
cKit	ATTGTGCTGGATGGATGGAT	GATCTGCTCTGCGTCCTGTT
Col1a1	GAGAGGTGAACAAGGTCCCG	AAACCTCTCTCGCCTCTTGC
Gata3	GCCTGCGGACTCTACCATAA	AGGATGTCCCTGCTCTCCTT
Gata4	CCATCTCGCCTCCAGAGT	CTGGAAGACACCCCAATCTC
Gata6	TACACAAGCGACCACCTCAG	TGTAGAGGCCGTCTTGACCT
Jnk1/Mapk	AGCAGAAGCAAACGTGACAAC	GCTGCACACACTATTCCTTGAG
Klf4	CCTCCCACGGCCCCCTTCAA	ATCTTGGGGCACATGCGCGG
Mdm2	TGTCTGTGTCTACCGAGGGTG	TCCAACGGACTTTAACAACTTCA
Mef2c	CGGTGTCGTCAGTTGTATGG	TGCAGTAGATGTGCGGCTTG
Nanog	AGGCTGCGGCTCACTTCCTTC	AGTCTGGCTGCCCCACATGGA
Nkx2.5	GGCTTTGTCCAGCTCCACT	CATTTTACCCGGGAGCCTAC
Oct4	CCAGGCAGGAGCACGAGTGG	GAGAACGCCCAGGGTGAGCC
Pdgfra	GGAACCTCAGAGAGAATCGGC	CATAGCTCCTGAGACCCGCTG
Pim1	CTGGAGTCGCAGTACCAGG	CAGTTCTCCCCAATCGGAAATC
Sma (Acta2)	GTACCACCATGTACCCAGGC	GCTGGAAGGTAGACAGCGAA
Stat3	CAATACCATTGACCTGCCGAT	GAGCGACTCAAACTGCCCT
Tgfb1	AGCTGCGCTTGCAGAGATTA	AGCCCTGTATTCCGTCTCCT
Trp53	GCGTAAACGCTTCGAGATGTT	TTTTTATGGCGGGAAGTAGACTG
